# Integrating tumor and healthy epithelium in a micro-physiology multi-compartment approach to study renal cell carcinoma pathophysiology

**DOI:** 10.1038/s41598-024-60164-w

**Published:** 2024-04-23

**Authors:** Maryna Somova, Stefan Simm, Adventina Padmyastuti, Jens Ehrhardt, Janosch Schoon, Ingmar Wolff, Martin Burchardt, Cindy Roennau, Pedro Caetano Pinto

**Affiliations:** 1 Department of Urology, University Medicine Greifswald, DZ7 J05.15, Fleischmannstraße 8, 17475 Greifswald, Germany; 2 Institute of Bioinformatics, University Medicine Greifswald, Fleischmannstraße 8, 17475 Greifswald, Germany; 3 Department of Obstetrics and Gynecology, University Medicine Greifswald, Fleischmannstraße 8, 17475 Greifswald, Germany; 4 Center for Orthopaedics, Trauma Surgery and Rehabilitation Medicine, University Medicine Greifswald, Fleichmannstraße 8, 17475 Greifswald, Germany; 5 Institute for Bioanalysis, Coburg University of Applied Sciences and Arts, Friedrich-Streib-Str. 2, 96450 Coburg, Germany

**Keywords:** Biotechnology, Urology, Renal cell carcinoma

## Abstract

The advent of micro-physiological systems (MPS) in biomedical research has enabled the introduction of more complex and relevant physiological into in vitro models. The recreation of complex morphological features in three-dimensional environments can recapitulate otherwise absent dynamic interactions in conventional models. In this study we developed an advanced in vitro Renal Cell Carcinoma (RCC) that mimics the interplay between healthy and malignant renal tissue. Based on the TissUse Humimic platform our model combines healthy renal proximal tubule epithelial cells (RPTEC) and RCC. Co-culturing reconstructed RPTEC tubules with RCC spheroids in a closed micro-perfused circuit resulted in significant phenotypical changes to the tubules. Expression of immune factors revealed that interleukin-8 (IL-8) and tumor necrosis factor-alfa (TNF-α) were upregulated in the non-malignant cells while neutrophil gelatinase-associated lipocalin (NGAL) was downregulated in both RCC and RPTEC. Metabolic analysis showed that RCC prompted a shift in the energy production of RPTEC tubules, inducing glycolysis, in a metabolic adaptation that likely supports RCC growth and immunogenicity. In contrast, RCC maintained stable metabolic activity, emphasizing their resilience to external factors. RNA-seq and biological process analysis of primary RTPTEC tubules demonstrated that the 3D tubular architecture and MPS conditions reverted cells to a predominant oxidative phosphorylate state, a departure from the glycolytic metabolism observed in 2D culture. This dynamic RCC co-culture model, approximates the physiology of healthy renal tubules to that of RCC, providing new insights into tumor-host interactions. Our approach can show that an RCC-MPS can expand the complexity and scope of pathophysiology and biomarker studies in kidney cancer research.

## Introduction

Renal cell carcinoma (RCC) is a urologic malignancy that originates in the renal tubular epithelium. It is the most prevalent type of kidney cancer and one of the 10 most common cancers diagnosed among men and women worldwide^1^. Current therapeutic approaches for RCC include robotic-assisted surgery and immunotherapy, both offering considerably favorable prognostic outcomes^2^. The introduction of immunotherapy, which leverages a patient's immune system to target and eliminate cancer cells significantly improved RCC clinical management^3^. Immune checkpoint inhibitors (ICI) are the most common immunotherapeutic agents and act by negating RCC cells the ability to evade immune cells^4^.

Early detection of RCC significantly impacts patient survival rates. The five-year survival rate exceeds 90% for patients with localized tumors at the time of diagnosis, whereas the survival rate is about 70% when tumors are detected to have metastasized to nearby tissues, such as the lymph nodes^5^. RCC originates from the renal proximal tubules when epithelial cells undergo trans-differentiation and acquire a distinct malignant phenotype^6^. The molecular triggers behind this process are still poorly understood, nonetheless, the loss of the von Hippel-Lindau (VHL) protein ligase activity is recognized as a hallmark of this transformation^7^. VHL is a mediator of the cellular response to hypoxia—low oxygen levels—and under normal physiological conditions, it facilitates the degradation of hypoxia-inducible factors (HIF). VHL is transiently inactivated with a low oxygen supply, enabling an enhanced HIF activity to counteract the effects of hypoxia. In RCC cells, VHL becomes constitutively inactive in the presence of physiological oxygen conditions, resulting in the accumulation and activation of HIF^8^. These events lead the reprogramming of energetic metabolism characteristic of RCC. The metabolic shifts are related to the grade of the tumor and are believed to progress with the disease^9,10^. Tumor cells require a multitude of nutrient to support their high rate of proliferation^11,12^, and RCC upregulate glucose uptake and metabolism to fuel a predominantly glycolytic activity. High levels of glycolytic enzymes are associated with poor patient survival, underscoring the impact of the metabolic shift in this type of cancer^13,14^. On the flip-side, mitochondrial bioenergetics is impaired, with a marked reduction of ATP production from oxidative phosphorylation (OxPhos)^15^. Moreover, to thrive in a nutrient-deficient environment RCC can accumulate lipids and alter fatty acid metabolism to meet their energetic needs^16,17^. These alterations collectively contribute to the characteristic angiogenic and immunogenic nature of the tumors. This altered metabolism has been implicated on the prolific infiltration of immune cells observed in RCC, as well as the regulation of angiogenesis and inflammatory signatures^18–20^.

The elevated secretion of angiogenic factors (e.g. vascular endothelial growth factors—VEGF) promotes the neo-vascularization of malignant tissue ensuring the necessary nutrient supplies to fuel growth^21^. The abundant release of immune factors (Interleukin 6 and 8—IL6, IL8) by RCC cells dampens the immune response against the tumor and recruits different populations of immune cells^22^. These cells infiltrate the tumor extracellular matrix (TEM), a complex hypoxic microenvironment rich in fibrotic tissue and vascular sprouts that encompasses both malignant and non-tumor cells, that support the activity and survival of cancer cells by further depressing the activity of potentially cytotoxic immune cells^23^. The composition of the TEM is believed to significantly affect responses to systemic therapy, namely ICI^24,25^.

During RCC development, cancer cells undergo different stages and can retain characteristics of non-tumor cells and tissues, that are beneficial to their survival such as glucose re-uptake and excretory activity^26^. Within the kidney, RCC are highly localized tumors with distinct morphological features, that share the blood supply with healthy tissue^27^.

Despite the advances in RCC treatment and the positive outcomes they offer, the detection of this type of cancer remains challenging. RCC remains asymptomatic in its early to mid-stages and renal function is typically not affected. Therefore, these cancers evade routine screening and renal function monitoring. As a consequence, most cases of RCC are discovered by accident when patients undergo imaging diagnostics for unrelated issues^6^. Moreover, to date, no qualified biomarkers have been identified for the detection of RCC. Collectively, these factors can contribute to delayed diagnosis and treatment initiation, and highlight the need for novel detection tools.

Our understanding of RCC pathophysiology and its underlying molecular mechanisms was significantly advanced by the widespread use of established cell lines in laboratory practice. Several models, such as the Caki-1, 786-O, and A-498 cells have contributed to unraveling the regulatory elements behind tumor hypoxia, adhesion, proliferation, and apoptosis^28^. Despite their valuable contributions, cell lines do not recapitulate the heterogeneity and complexity of native tumors. In general, cell lines are prone to genetic instability during prolonged culture, deviating from their initial phenotypes and losing key tumor characteristics. In conventional cultures, cells lack the tumor microenvironment and dynamic interactions with different cellular components^29^. Most cell lines were isolated from aggressive tumor types and selected for their proliferative nature in culture, poorly representing different tumor types. The use of hydrogel cultures, enables cells to grow and differentiate in a 3D matrix, typically as spheroids^30^. These cultures also introduce more heterogenicity to the tumor phenotype, thanks to the spatial organization of the cells, with the inner and outer regions of the spheroids experiencing differential nutrient supplies and adhesion properties. The physiology of 3D-RCC constructs can be further extended with the use of xenograph animal models, where tumor cells can tap into the vasculature and an on-demand nutrient supply^31^. However, these models are typically based on immune-deficient rodents that have limited human translation and require extensive experimental work. These studies are also limited by the number of animals allocated per study and the humane-end point, required to minimize animal use and discomfort^23^.

Microphysiological systems (MPS) have emerged as a promising substitute for animal studies, particularly in research demanding strong human translational relevance, thanks to their ability to recreate specific elements of human physiology. MPS introduce dynamic stimuli to cell cultures and offer a diverse array of formats and platforms, ranging from off-the-shelf models to customized designs. These systems enable the culture of complex structures like spheroids with the incorporation of continuous or recirculating flow, and can subject cells to mechanical stimuli such as compression and shear stress, in a controlled manner enhances experimental accuracy. Moreover, multi-compartment systems allow the co-culture of different cell types sharing the same circulation and the generation of increasingly complex models^32^. Recent efforts in the development and characterization of renal-MPS have demonstrated that fully functional, polarized, and compartmentalized renal proximal tubules, with secretory functions can be recreated in vitro^33^. Initial studies have also shown that the angiogenic nature of RCC can effectively be recapitulated in vitro, with the neo-vascularization of a matrix embedded with endothelial cells promoted by RCC secretions^34^.

Employing MPS can increase the complexity of RCC models in vitro, expanding the phenotype of tumor cells beyond conventional 3D spheroid culture and incorporating additional elements of renal physiology. In this study, we have established an advanced RCC model that incorporates both healthy renal tubules and RCC cells, interconnected via a shared perfusion circuit with two separate culture chambers. The renal tubule component is based on the RPTEC-TERT1 cell line and was engineered using a custom 3D-printed chamber, enabling the formation of a self-assembled renal tubule embedded in a collagen I matrix^35^. For the RCC component, CAKI-1 cells spheroids embedded in an agar-collagen hydrogel were used. Based on the TissUse Humimic platform^36^, the primary objective of this approach is to investigate the interactions between healthy and tumor epithelium and explore the effects of RCC secretions.

## Results

### Establishment of the micro-perfused 3D-RCC model

**Figure 1:**
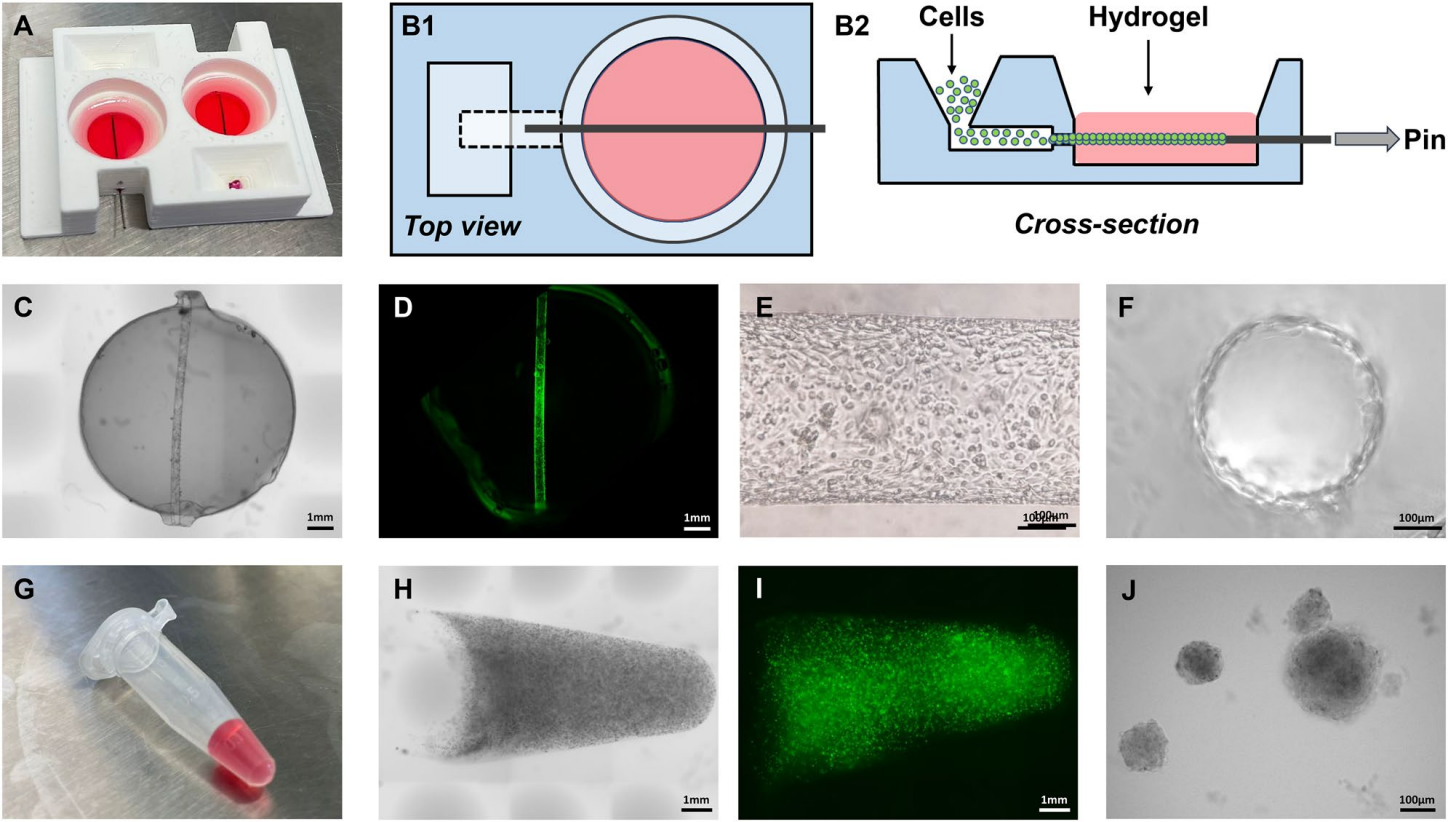
3-Dimetional cell culture formats. **(A)** A 3D-printed construct comprising 2 culture chambers for the generation of self-assembled RPTEC tubules. **(B1-2)** Schematic representation of the culture chamber depicting the insertion of RPTEC cells into the collagen matrix. Negative pressure induced by pin extraction enables cell movement and adhesion to the hollow tube surface. **(C,D)** Disc-shaped gel with a 3D arrangement of RPTEC tubule, visualized under light microscopy and fluorescent microscopy (2× magnification). **(E,F)**. Detailed morphology of the renal tubule and its cross-section (10× magnification). **(G)** Agarose hydrogel used to generate CAKI-1 RCC-spheroids in a 1.5mL microcentrifuge tube. (**H,I)** Cone-shaped hydrogel populated with RCC spheroids, visualized under light microscopy and fluorescent microscopy (2× magnification). **(J)** Detail of the RCC spheroid morphology (10× magnification).

**Figure 2:**
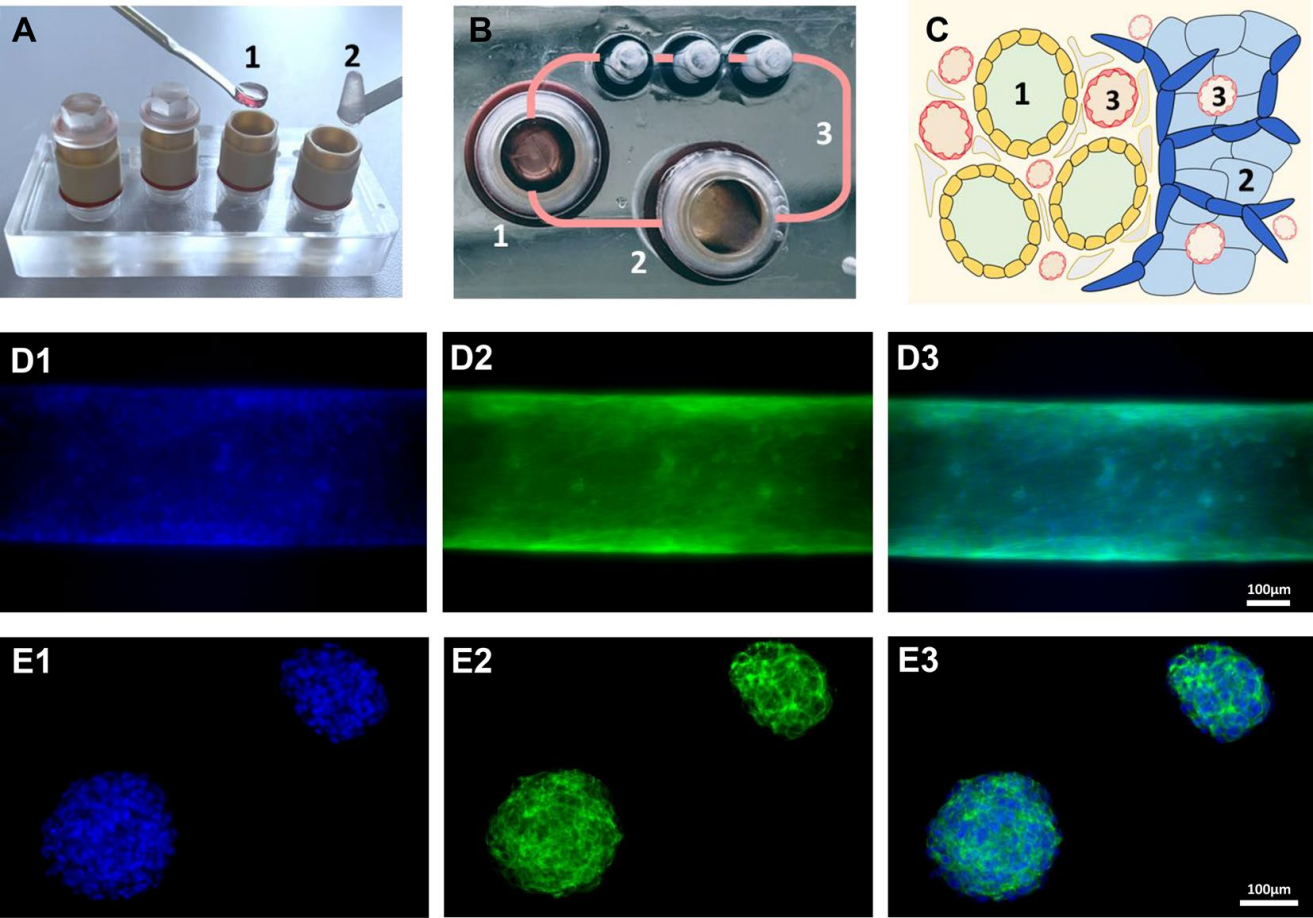
Microfluidic RCC model. The Humimic chip2 comprises 2 independent culture circuits accessible by 2 culture compartments each. In the co-culture set-up of the system a renal tubule embedded in a collagen disc (1), and an agar hydrogel with RCC spheroids (2) are transferred to the culture of the chip **(A)**. The microfluidic circuit re-circulates culture media **(B)** between the two compartments mimicking the circulation (3) between cancer and healthy epithelium **(C)**. The nuclear marker Hoechst33342 and cytoskeleton staining Phalloidin488 were used to highlight the morphology of the RPTEC tubules **(D1-3)** and RCC spheroids **(E1-3).**

To recreate a spatially complex environment that better represents RCC physiologically, our approach integrates both healthy epithelial renal cells (RPTEC) and renal cancer cells (RCC-Caki-1) in the TissUse Humimic Chip2 MPS. This micro-perfusion platform generates recirculating flow between two independent culture compartments using peristaltic pulsation. Renal proximal tubules, representing the healthy renal epithelial, were reconstructed using bespoke 3D-printed culture chambers (Fig. 1A, B1-2). These chambers were designed to generate a self-assembled RPTEC-TERT1 renal tubule, embedded in a cross-linked collagen type I matrix. The resulting matrix consists of an 8 mm by 3 mm disc, with approximately 150 µL in volume, comprising a renal tubule, with a length of 8mm and a cross-section of 0.4 mm (Fig. 1C–F; Fig. 2 D1-3). RCC spheroids derived from CAKI-1 cells were generated using an agar/collagen type I matrix, polymerized in a 1.5 mL microcentrifuge to obtain a conical shape with a volume of 200µL. CAKI-1 developed into non-hollow spheroids with approximately 50 µm in diameter and consisting of about 40–50 cells (Fig. 1G–J; Fig. 2E1-3). Both the renal tubule and RCC matrix constructs are compatible with the Humimic Chip2 culture compartments (Fig. 2A, B) and were optimized to maintain their structural integrity under the sheer stresses derived from the pulsating flow. Both RPTEC-TERT1 cell and CAKI-1 cells maintain their viability under the dynamic culture conditions used in the experimental set up (Supplementary Fig. 2).

**Figure 3:**
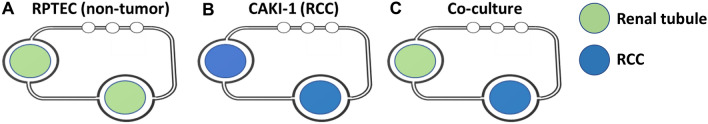
Schematic representation of the experimental set-up. **(A)** RPTEC tubules in single culture. **(B)** RCC spheroids in single culture. **(C)** Co-culture of RPTEC tubules and RCC spheroids.

In the kidney, RCC develops into a tumor mass with defined well-boundaries separating it from the healthy renal epithelium, with a common vasculature (Fig. 2C). Our model mimics the RCC physiology by incorporating malignant and non-malignant cells that populate different niches and share the same culture media, recirculating between them. This system enables interactions between the different cell types to take place and can recapitulate aspects of intricate RCC microenvironment, absent in conventional in vitro models.

### Dynamic RCC cell culture deregulates the expression of renal markers

The expression of 14 genes, recognized as relevant markers of renal proximal tubule physiology, was analyzed to determine the impact of co-culturing healthy renal tubules with RCC spheroids under dynamic conditions. Changes in gene expression patterns were evaluated relative to single culture conditions, where only renal tubules or RCC spheroids were kept in MPS culture.

**Figure 4:**
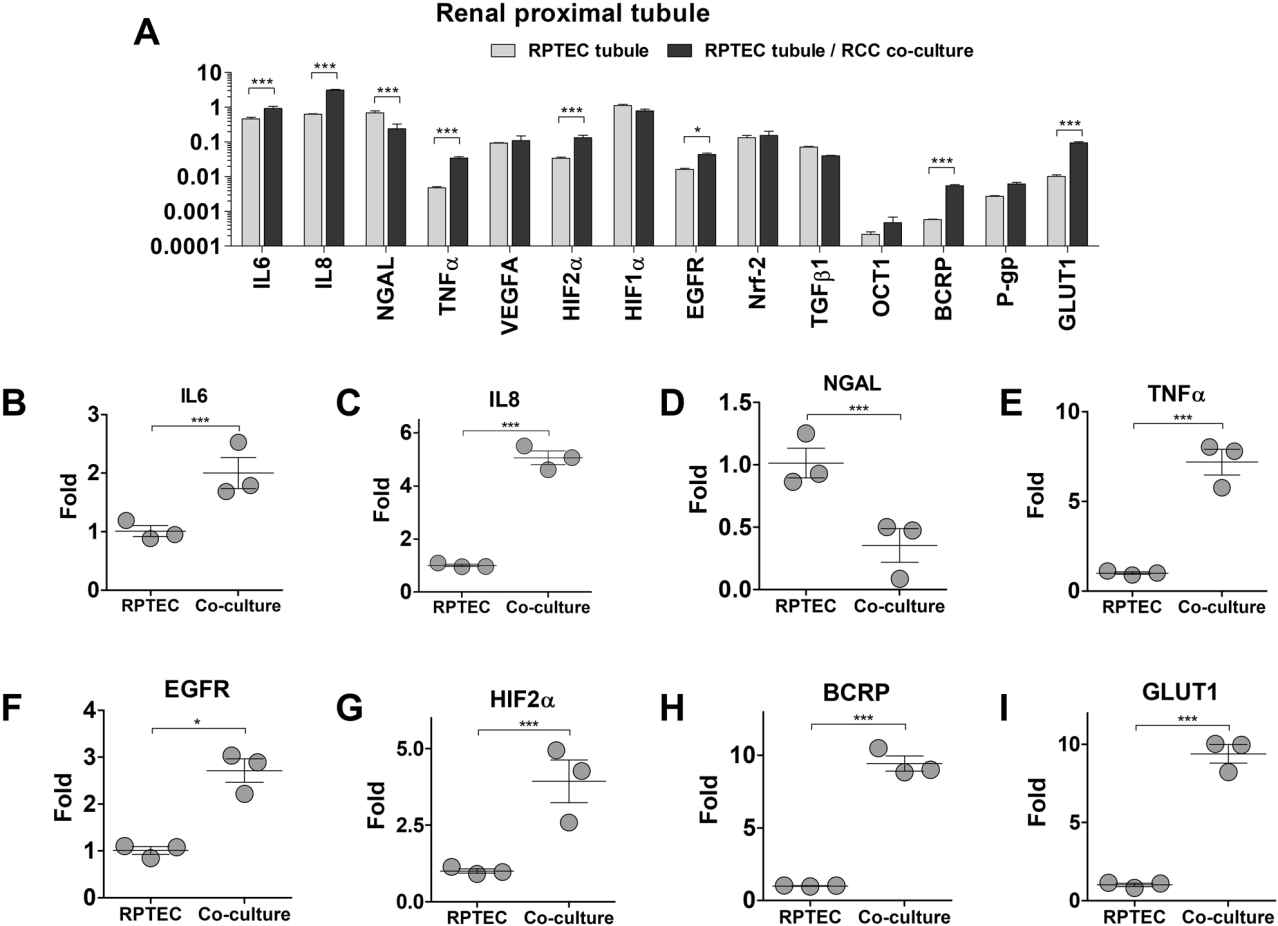
Differences in gene expression of RPTEC tubules, co-cultured with RCC spheroids. **(A)** Absolute expression values (2−ΔCt), presented in a log10 scale. Significant statistical differences were found in the expression of IL-6 **(B)**, IL-8 **(C)**, NGAL (D), TNF **(E)**, EGFR **(F)**, HIF2A **(G)**, BCRP **(H)**, and GLUT1 **(I)** Data represents the analysis of three independent samples. Statistical significances were determined using a two-tailed unpaired t-test (*p < 0.05; **p < 0.01; ***p < 0.001).

In RPTEC, the expression of markers was substantially impacted by the dynamic co-culture with RCC spheroids (Fig. 4A). The levels of immune factors Interleukin-6 and 8 (IL-6 and IL-8) and Tumor Necrosis Factor alfa (TNF-α) were significantly upregulated to 2.0 ± 0.5-fold, 5.0 ± 0.5-fold, and 7.2 ± 1.2-fold, respectively. Interestingly, the innate immune factor Neutrophil Gelatinase-associated Lipocalin (NGAL), was the only downregulated marker observed (0.35 ± 0.23-fold), in our analysis (Fig. 4B–E). The expression of Epidermal Growth Factor Receptor (EGFR), a key functional regulator of RPTEC, was elevated by 2.7 ± 0.4-fold. The expression of Hypoxia Inducible Factor-2-alfa (HIF-2α), a key regulator in RCC development, was increased by 3.9 ± 1.2-fold. The efflux drug transporter Breast Cancer Resistance Protein (BCRP), associated with resistance to chemotherapeutical agents, was upregulated by 9.4 ± 0.9-fold. Expression of the Glucose Uptake Transporter 1 (GLUT1) was also upregulated (9.4 ± 1.0-fold), further highlighting the impact of RCC on the function of non-malignant tubular cells (Fig. 4F–I). Noteworthy is also the fact that the expressions of Vascular Endothelial Growth Factor (VEGF) and Hypoxia Inducible Factor-1 (HIF-1) were not altered by RCC co-culture. Both VEGF and HIF-1 play important roles in the onset of angiogenesis of metabolic shifts in RCC.

**Figure 5:**
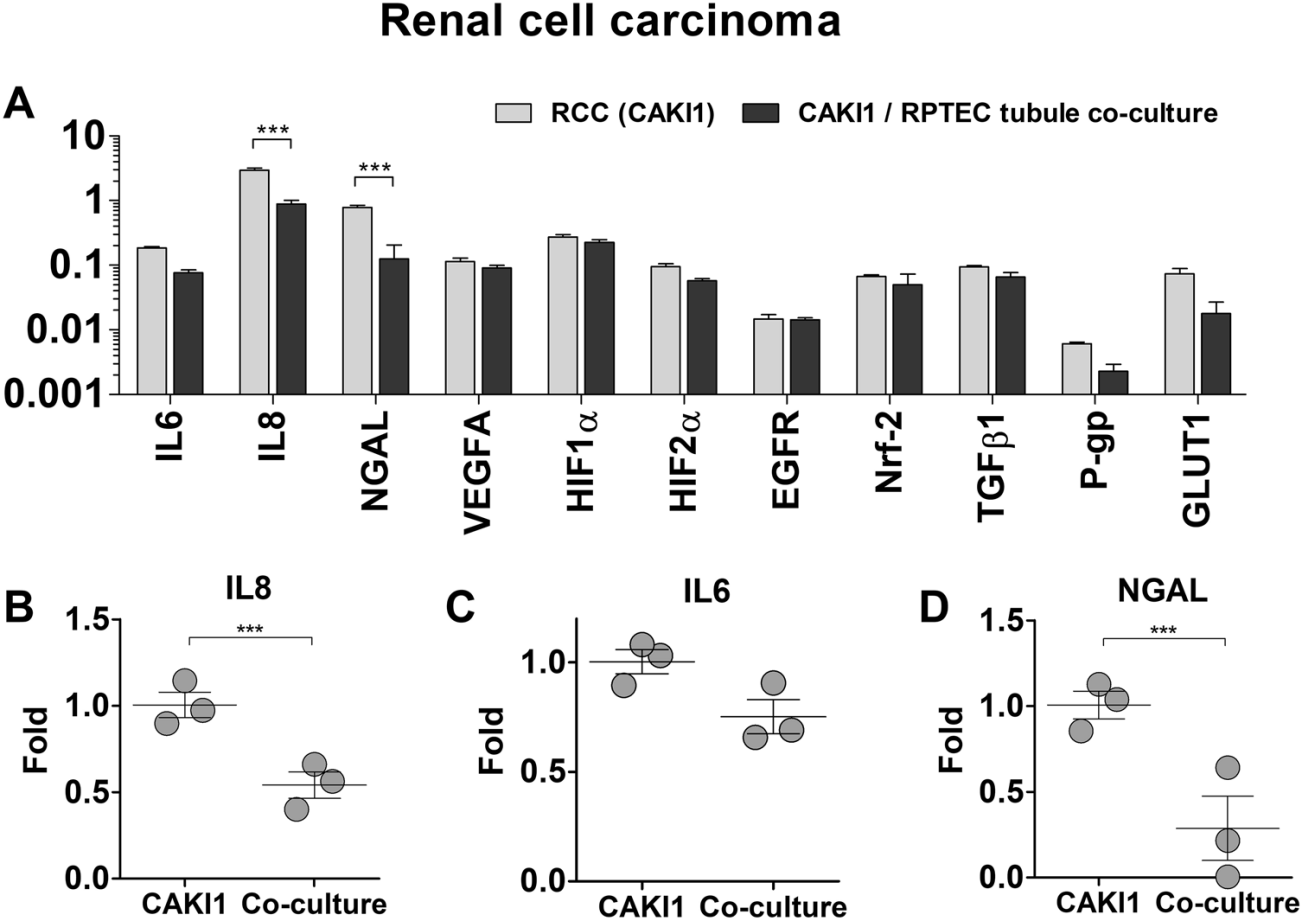
Differences in gene expression of RCC spheroids, co-cultured with RPTEC tubules. **(A)** Absolute expression values (2-ΔCt), presented in a log10 scale. Significant statistical differences were found in the expression of IL-6 **(B)**, IL-8 **(C)**, and NGAL **(D)**. Statistical significances were determined using a two-tailed unpaired t-test (*p < 0.05; **p < 0.01; ***p < 0.001).

In RCC, changes in marker expression were not extensive, highlighting the fact that the tumor cells are not substantially affected by co-culture with non-tumor proximal tubule cells (Fig. 5A). The levels of IL-6 and IL-8 were downregulated to 0.75 ± 0.14-fold and 0.54 ± 0.13-fold, respectively. The expression of TNF-α was not detected in RCC spheroids. NGAL expression was also significantly deregulated, to 0.29 ± 0.32-fold, similar to the behavior observed in non-tumor cells (Fig. 5B–D). Interestingly, the expressions of P-glycoprotein (P-gp) and GLUT1, were reduced to 0.68 ± 0.28-fold and 0.45 ± 0.33-fold. The levels of VEGF, HIF-1, and HIF-2α were not altered, further reiterating the stability of the RCC spheroids. The differential expression of markers experienced by RPTEC under the influence of RCC unveils the impact that malignant cells exert on the phenotype of healthy epithelium. The expressions of Nuclear factor erythroid 2-related factor 2 (Nrf2) and Transforming growth factor-beta 1 (TGFβ-1) were not altered in non-tumor cells. This fact indicates that processes involving cell proliferation associated with RCC are likely still muted under the experimental conditions tested.

### Secretion of immune factors and lactate consumption

**Figure 6:**
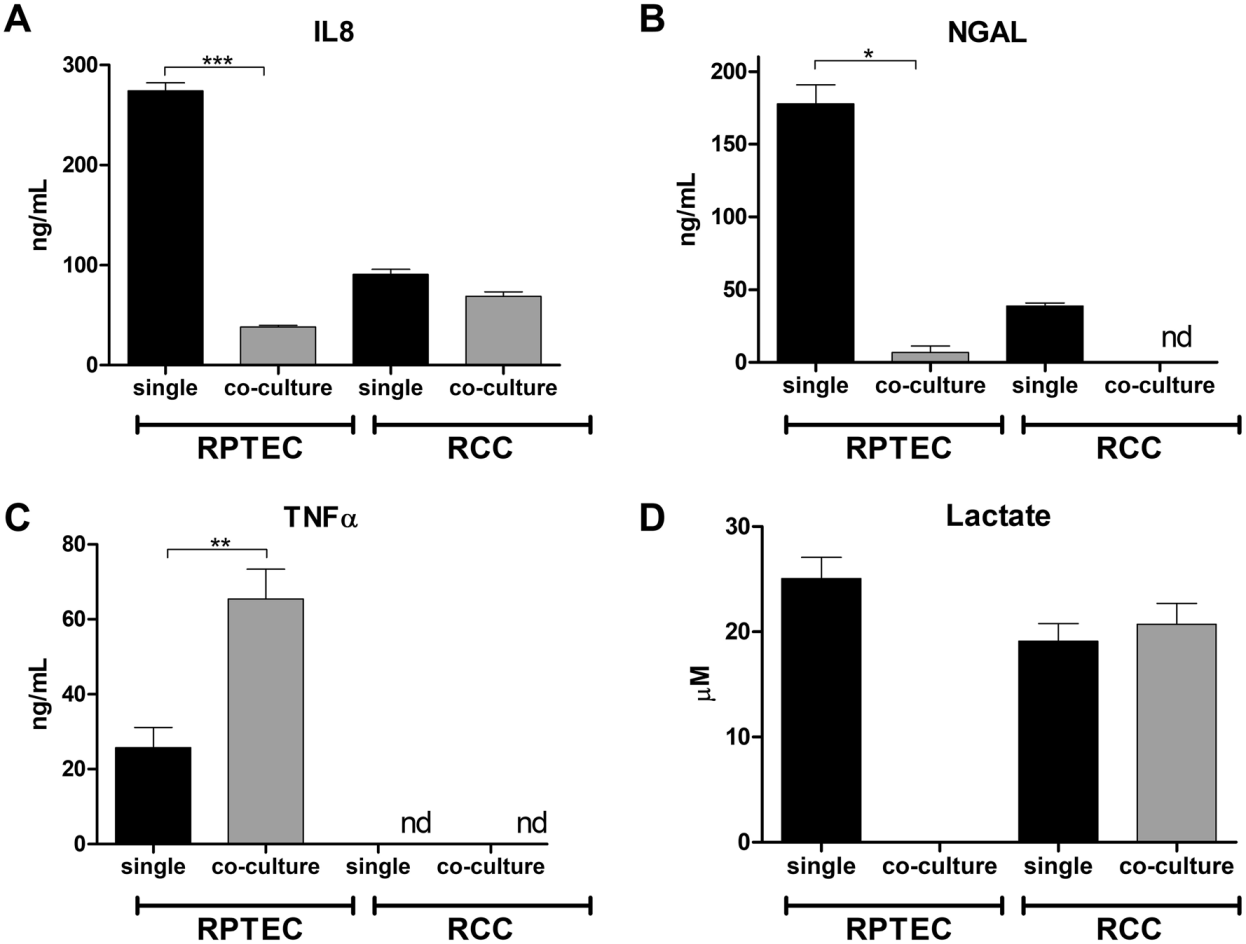
Extracellular secretion of immune factors and lactate. The secretion of IL-8 **(A)**, NGAL **(B)**, TNF **(C),** and lactate **(D)** was evaluated in single and co-cultures of RPTEC tubules and RCC spheroids. The data described represents samples collected from a minimum of three independent experiments, where supernatant was collected from all the culture compartments of the Humimic chip. Statistical significances were determined using a two-tailed unpaired t-test (*p < 0.05; **p < 0.01; ***p < 0.001).

The analysis of RCC-associated immune factors present in the supernatant of the MPS was performed to further investigate the impact of co-culture dynamic conditions on cellular immunogenicity. The levels of lactate were evaluated as an indication of metabolic activity (Fig. 6). The secretion of IL-8 by RPTEC was reduced by approximately sevenfold under the effect of RCC co-culture. The IL-8 levels under co-culture conditions are consistent with the levels observed in RCC single culture (Fig. 6A). The levels of NGAL were reduced by 26-fold in co-culture conditions relative to RPTEC tubule single culture. NGAL released by RCC was limited and seemingly precluded in the presence of the non-malignant RPTEC tubule (Fig. 6B). The levels of extracellular TNF-α secreted by RPTEC were elevated by 2.5-fold under co-culture conditions. The RCC spheroids did not express or secrete TNF-α. Expression of IL-6 was also evaluated, but no levels of this immune factor were detected across all conditions (Fig. 6C).

Extracellular lactate analysis showed that in single culture, both RCC spheroids and RPTEC tubules release similar levels of this metabolite. Interestingly, in the co-culture set-up, no lactate was detected in the RPTEC tubule compartment, while the RCC compartment maintained its lactate levels. Since the culture media and subsequent cellular secretions are shared between both compartments, the absence of lactate indicates that RPTEC are consuming the lactate produced in the system (Fig. 6D). This fact also shows that, in co-culture, the RPTEC tubule and RCC spheroids maintain their own microenvironments and are capable of regulating the secretion and consumption of compounds independently of their shared perfusion.

### RCC impacts the metabolic activity of healthy RPTEC

**Figure 7:**
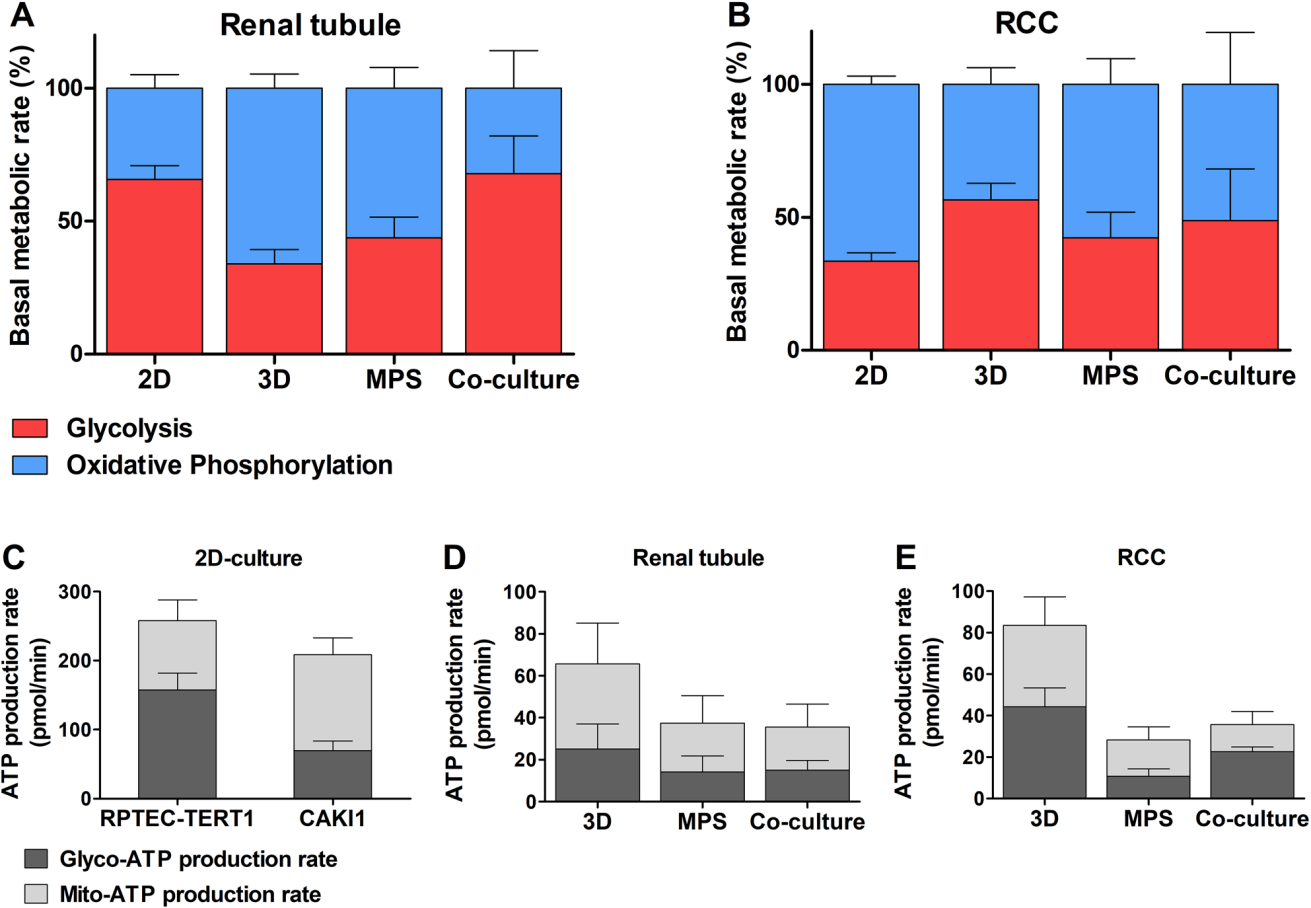
Metabolic activity analysis based on the oxygen consumption rate (OCR) and extracellular acidification rate (ECAR). Basal metabolic rate in the RPTEC tubule **(A)** and RCC spheroids **(B)**. ATP production rate in 2D-cultures **(C)**, Renal tubules **(D)**, and RCC spheroids. Statistical significances were determined using a two-tailed unpaired t-test (Supplementary Table 5).

The Seahorse XF is a powerful tool used to evaluate cellular bioenergetics by measuring oxygen consumption rate and extracellular acidification rate in real time. Using this platform, we determined the impact of RCC co-culture on the cellular respiration and glycolytic activity of non-malignant RPTEC (Fig. 7).

RPTEC cultured in conventional 2D format demonstrated a predominant glycolytic phenotype (66 ± 5%). In our static 3D format, RPTEC tubules reduced their dependence on glycolysis to (34 ± 5%). The predominant OxPhos phenotype is maintained when the RPTEC tubules are subjected to MPS culture (glycolysis:44 ± 8%; OxPhos:56 ± 8%). This fact shows that recreating a 3D tubular architecture plays a major role in the recovery of the physiological metabolic activity of renal proximal tubules, relative to micro-fluidic culture conditions. When the RPTEC are co-culture with RCC, their metabolism reverts to a state where glycolysis is predominant (glycolysis:68 ± 14%; OxPhos:32 ± 14%; Fig. 7A,D).

RCC cells, interestingly, reveal a predominately OxPhos metabolism when cultured in conventional 2D conditions, with glycolysis representing about 34% of their overall energetic output. In static 3D culture, RCC cells shifted towards a more balanced metabolism with similar glycolytic (57 ± 6%) and OxPhos (44 ± 6%) components. The introduction of dynamic MPS culture and subsequently co-culture conditions did not substantially alter the metabolic activity of RCC observed under 3D static conditions (Fig. 7B,E). These findings highlight the stability of energy production in RCC and their capability of promoting a metabolic shift towards glycolysis in non-tumor RPTEC tubules. Statistical analysis of the metabolic activity is described in supplementary information Table 5.

### Impact of microfluidic and tubular architecture on RPTEC phenotype

**Figure 8:**
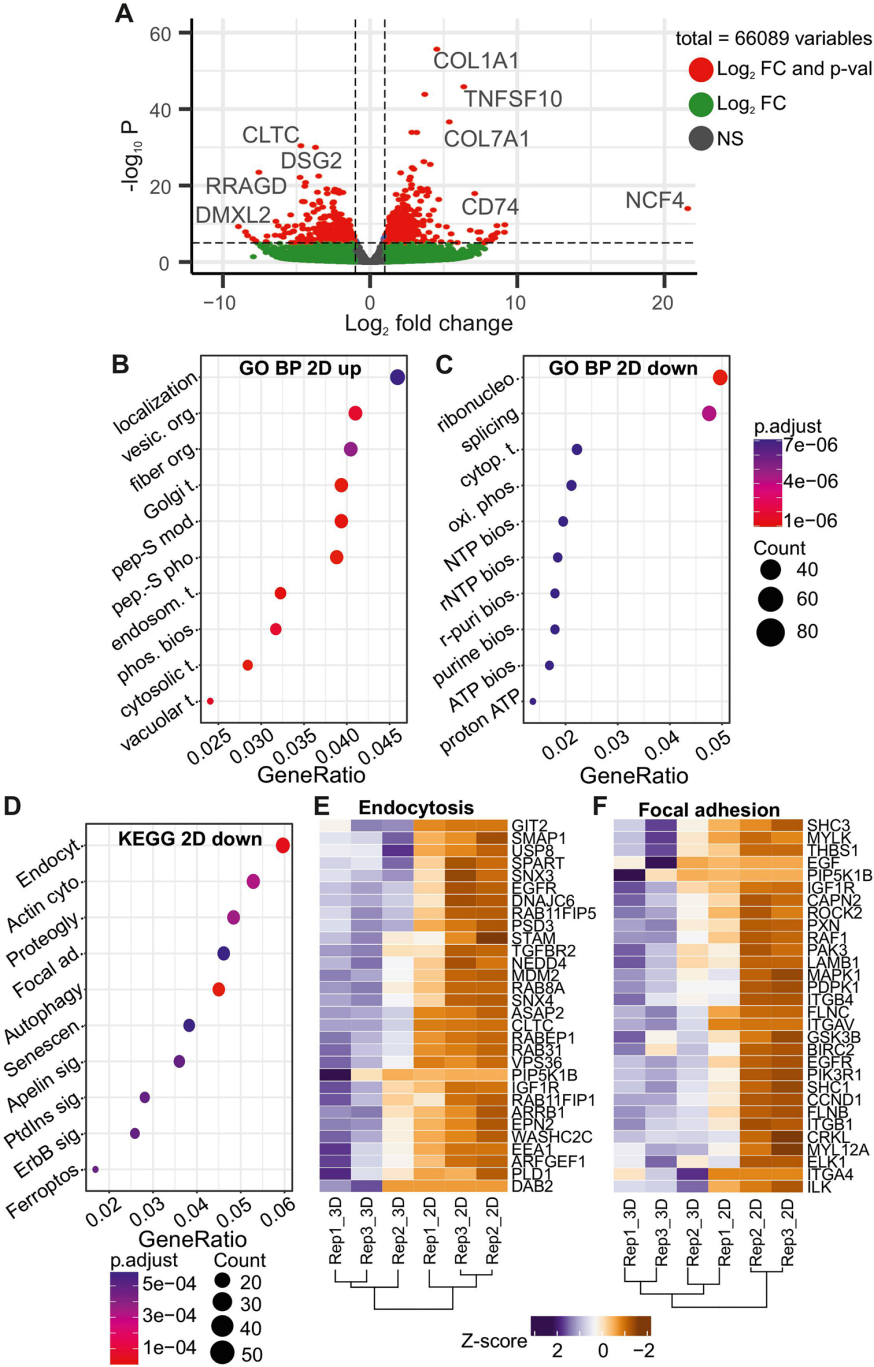
Differential expression and overrepresentation analysis between 2 and 3D characterizing functional discrepancies. Overall, DEGs based on the FC and adjusted p-value differences are plot **(A)**, and significantly down- (B) and up-regulated **(C)** DEGs are used to identify overrepresented “biological process” in the GO as well as most affected KEGG pathways **(D)**. Main pathways Endocytosis **(E)** and Focal adhesion **(F)** and their expression profile for the top30 DEGs are visualized via heatmaps. Abbreviations in dot plots: localization…establishment of protein localization to organelle; vesic. org…vesicle organization; fiber org…regulation of supramolecular fiber organization; Golgi t…Golgi vesicle transport; pep-S mod…peptidyl-serine modification; pep. S pho…peptidyl-serine phosphorylation; endosome. t…endosomal transport; phos. bios…phospoholipid biosynthetic process; cytosolic t…cytosolic transport; vacuolar t…vacuolar transport; ribonucleo…ribonucleoprotein complex biogenesis; splicing…RNA splicing; cytop. t…cytoplasmic translation; oxi. phos…oxidative phosphorylation; NTP bios…NTP biosynthetic process; rNTP bios…rNTP biosynthetic process; r-puri bios…purine rNTP biosynthetic process; purine bios…purine NTP biosynthetic process; ATP bios…ATP biosynthetic process; proton ATP…proton motive force-driven ATP synthesis.

To further characterize the impact of dynamic MPS culture and the reconstruction of a 3D tubular architecture on the phenotype of RPTEC in vitro, we compared the global gene expression of primary RPTEC culture in conventional 2D-format and MPS, using Next generation sequencing (RNA-Seq). Differential expression analysis between 2D- and 3D-format were performed identifying 4432 differentially expressed genes (DEGs). From these, 1944 DEGs were down- and 2488 DEGs upregulated in the 2D format (Fig. 8A). Gene set overrepresentation analysis on the biological processes of the Gene Ontology (GO) and Kyoto Encyclopedia of Genes and Genomes (KEGG) functional hierarchies was performed.

Relative to their expression in RPTEC tubules culture in MPS, the top10 significantly enriched biological processes (BP) in 2D-culture involve the organization and movement to intracellular vesicles (e.g.: “vesicle organization”, “endosomal transport”, “vacuolar transport”, “cytosolic transport”). On the other hand, 2D-RPTEC downregulated genes showed enrichment in the BPs: “transcription and protein translation”, “oxidative phosphorylation”, “ATP biosynthesis”, and “purine biosynthesis” (Fig. 8 B,C). Further, the KEGG functional hierarchy analysis shows that “endocytosis” and “focal adhesion”, among others, are enriched under downregulation in 2D-RPTEC (Fig. 8D). These functional analyses demonstrate the importance of RPTEC in MPS culture by the range of DEGs and regulatory changes of biological processes and pathways in 2D. Across the three biological replicates (2D and 3D each) the expression of DEGs associated with “endocytosis” and “focal adhesion” is consistently downregulated with only slight changes (Fig. 8E,F).

**Figure 9:**
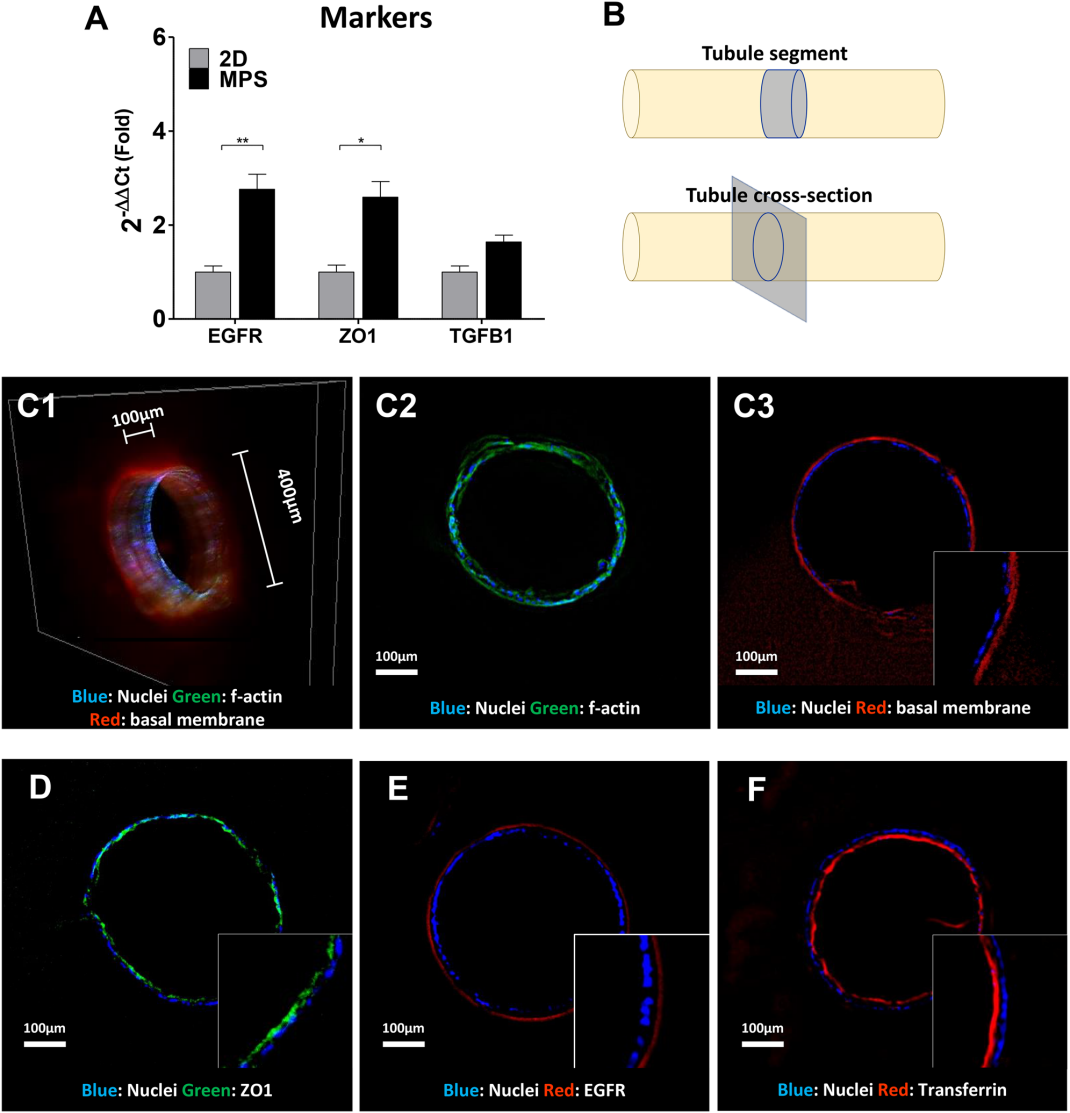
Validation of renal markers expression in dynamic culture and morphological characterization of the renal tubules. The expression of the DEGs EGFR, ZO1 and TGF-β, identified in the RNA-Seq analysis, were confirmed upregulated in RPTEC-TERT1 via qPCR **(A).** Depiction of the segment and tubule cross-section used for morphological immunofluorescent characterization **(B)**. 3-dimentioal projection of a tubule segment 100 µm in length (C1), Cross-sections of f-filamentous stained with Phalloidin-488 **(C2)** and basal membrane stained with PNA-555 **(C3)**. ZO1 can be observed in the apical membrane of the tubule **(D)**, EGFR is expressed on the basolateral side **(E)**, and Transferrin-555 accumulated on the apical side of the tubule **(F)**.

The qPCR analysis of EGRF, Zonula Occludens-1 (ZO1), and TGF-β1 revealed that these DEGs identified in RNA-Seq are also upregulate in RPTEC-TERT1 cells. The EGFR is a known binding side for receptor mediated endocytosis (Fig. 9A). Enhanced ZO1 expression promotes cell polarity which facilitates endocytic processes. TGF-β cytokines are promotors of clathrin-dependant endocytosis^37,38^. Immunofluorescent staining of the tubular cross-section provides an adequate characterization of the renal tubule morphology and its polarity, with clear differentiation between the basolateral and apical membranes (Fig. 9B). The tubule profile is defined by a F-actin staining (Phalloidin, Fig. 9C-2), and peanut agglutinin (PNA) lectin staining highlighted the presence of glycosylated proteins in the outer region of the tubule, forming a defined basal membrane (Fig. 9C-2). ZO1 is localized in the apical membrane, facing the tubule lumen (Fig. 9D) and EGFR is localized in the basolateral membrane, facing the tubule exterior (Fig. 9E). The proper physiological localization of these markers underscores the polarized nature of the reconstructed tubules. Transferrin is incorporate in the proximal tubules via endocytosis, and can be seem accumulating in the apical membrane (Fig. 9F).

## Discussion

The implementation of MPS in biomedical research is enabling an increase in the biological complexity of in vitro systems. Without the constraints of growing solely in flat surfaces or static environments, micro-perfused platforms can recapitulate important morphological features. Recently, MPS have also been employed to explore the dynamics of combining different organ components, using multi-compartment platforms^39^. Recapitulating the physiology of the renal proximal tubules in MPS has been the subject of extensive research mainly directed at drug development applications, offering valuable insights about pre-clinical renal safety, pharmacokinetics, and pharmacodynamics^40^. Nonetheless, the use of complex RCC models in experimental urology, drug testing, and biomarker discovery is still limited^34^. In the present study, we take advantage of the progress in renal-MPS models to develop a complex in vitro RCC model that mimics the interaction between healthy and malignant renal tissue.

The TissUse Humimic chip2 MPS was selected as the basis of our model because it provides a controllable and stable re-circulating micro-perfusion (Fig. 2A,B). Moreover, the system offers easy access for the collection of supernatant and cells over time and at a prespecified end-point. 3D-printed culture chambers represented a cost-effective and flexible tool to optimize and scale up the generation of reconstructed proximal tubules. Genipin and NaOH were used to cross-link the collagen matrices and improve their stiffness^33^. This facilitated handling, prevented the tubules from collapsing, and enabled the collagen matrices to tolerate the pulsating perfusion cycles maintaining their structural integrity (Fig. 2D1-2). Similarly, the agar hydrogel fulfilled the same purposes and also ensured a homogeneous CAKI-1 spheroid formation, while arguably limiting their proliferation and size. Under these previously optimized conditions to generate cancer-spheroids^41^, CAKI-1 cells developed into relatively small rounded non-hollow structures (Fig. 2E1-3). Collagen was not used to embed CAKI-1 spheroids since the high concentration of NaOH used to polymerize the matrices was toxic to the cells. A reconstructed contiguous tubule was chosen as the model for healthy renal epithelium, considering that previous research has shown that a stiff, anisotropic extracellular matrix with a curvature represents an optimal template for RPTEC differentiation^42^. This microenvironment reinforces epithelial cellular morphology, augments the expression of key renal markers, and maximizes physiological functions in vitro^43^. The common culture media circulation between the independent culture compartments exposed the renal tubules to RCC secretions, and vice-versa. Different perfusion set-ups (Fig. 3) enabled the comparison between the presence or absence of RCC on the expression profile and activity of the non-tumor renal tubule.

Co-culture of the renal tubules with RCC results in substantial deregulation of the phenotypical markers analyzed (Fig. 4). IL-6 and IL-8 are cytokines released by RCC that contribute to tumor progression, angiogenesis, and the maintenance of an inflammatory microenvironment^22^. At the gene level, IL-8 expression is upregulated in the renal tubules, while its secretion is substantially reduced. Interestingly, IL-8 levels in co-culture with RCC are consistent with the levels observed in single RCC culture (Fig. 6). These contradictory findings indicate that RCC may indirectly regulate IL-8 release by RPTEC, bringing its secretion to levels suited to the RCC microenvironment, when sharing the same circulation. Secretion of IL-6 was not detected across all conditions. This fact may be explained by the short half-life of this cytokine and its stability in the circulating media, which is re-perfused for 5-days^44^. The substantial reduction in the expression and secretion of NGAL in both the renal tubules and RCC in co-culture highlights the role of this innate immune factor in RCC pathophysiology. NGAL is a well-characterized renal injury biomarker, where elevated levels in urine reveal acute kidney damage. On the other hand, its potential use to detect RCC is controversial, with limited research exploring the role of NGAL in RCC and its release by cancer cells^45^. TNF-α is another RCC-associated pro-inflammatory cytokine that also plays a role in tumor cell survival and proliferation, among other processes^46^. The expression and secretion of TNF-α is upregulated in the renal tubules exposed to RCC and, interestingly, this cytokine is not expressed by CAKI-1 cells. These findings show that RCC exerts a significant impact on the release of immune factors by healthy renal cells. These findings may, arguably, promote an immunogenic microenvironment surrounding non-tumor epithelium that benefits the survival of adjacent RCC cells. Moreover, these effects are compounded by the upregulation of genes involved in cell proliferation (EGFR), metabolism (HIF2A, GLUT1), and drug resistance (BCRP) in the renal tubules. Concurrently, the expression of renal markers and secretion of immune factors in RCC spheroids is relatively unaltered, except for NGAL (Fig. 5). These seemingly stable traits highlight the stability of the RCC phenotype in our models and its resilience to external factors.

The secretion of lactate further underlines the capability of both the renal tubules and RCC spheroids to maintain their separate microenvironments while sharing a common circulation (Fig. 6D). In the co-culture set-up, lactate levels were expected to increase, derived from the release by both cellular components. However, lactate is not observed in the renal tubule compartment, an indication that RPTEC are consuming the metabolite. Lactate is a bi-product of glucose metabolism and its secretion is indicative of glycolytic activity in the cells^47^. When exposed to RCC, the renal tubules likely resort to gluconeogenesis, a process that converts lactate into glucose, to fuel ATP production via glycolysis^48^. Metabolic activity analysis showed that the renal tubule, under dynamic culture conditions, relies predominately on mitochondrial respiration, a more energy-efficient ATP production process (Fig. 7). The introduction of RCC in dynamic culture pushes the renal tubule metabolism towards glycolysis. This fact accounts for the consumption of lactate by RPTEC observed, which uses the metabolic to increase their glycolytic output. On the other hand, MPS and co-culture conditions did not substantially alter the metabolic activity of RCC cells, while maintaining a balance between glycolysis and OxPhos. RCC seems to exert a significant effect on the energy production of RPTEC. Malignant cells seem to align the metabolism of healthy renal cells with their more glycolytic phenotype. Physiologically, RPTEC mainly produce ATP via OxPhos while RCC are predominately glycolytic. While glycolysis is an ineffective process relative to OxPhos, it supports the RCC growth and immunogenicity^49^. This metabolic pathway provides anabolic substrates that support cell proliferation and migration, and the acidification of the extracellular microenvironment^50^. Overall, these findings show that RCC can dictate the homeostasis of RPTEC in their vicinity, under the dynamic culture conditions selected for this study.

The profound phenotypical changes that a 3D tubular architecture combined with MPS conditions promotes in the phenotype of healthy renal epithelial cells was evident from the RNA-seq analysis of human primary RPTEC cultures (Fig. 8). These cells were chosen for high-content comparison since they are known to retain better phenotypical features than immortalized human RPTEC, hence providing a more physiologically relevant starting point^51^. Our analysis shows that RPTEC culture in 2D conventional culture retains a high activity of cytoplasmatic transport and phospholipid biosynthesis likely associated with cellular growth. On the other hand, two BP analysis showed that in 2D, OxPhos and related ATP production processes downregulated, corroborating the observation that RPTEC in 2D have a reduced OxPhos activity and rely predominately on glycolysis for energy production (Fig. 7A). This previously documented phenomenon^52^, where RPTEC shift their metabolism towards glycolysis when maintained in conventional cultures, despite the abundance of atmospheric oxygen, is allegedly due to the enhanced proliferation in 2D and the abundance of glucose in most culture mediums used. Our study demonstrates that a 3D tubular architecture, independent of the glucose concentrations in the media and microfluid conditions, can revert the metabolism of RPTEC to a predominant OxPhos state. In 2D-RPTEC mechanisms associated with a well-defined basolateral and apical cell polarization such as focal adhesion and endocytosis are also repressed, further highlight the impact of the dynamic culture in the morphology and activity of RPTEC^38^. Morphological analysis (Fig. 9) confirms that the reconstructed tubules have a physiological polarization, with defined basolateral and apical membranes. An enhancement is focal adhesion is evident from the organized and contiguous basement membrane surrounding the tubule. Endocytic activity observed with the accumulation of transferrin, a carrier protein recovered by the proximal tubule via endocytosis^53^, in the luminal side.

In this study, we demonstrate that co-culturing healthy renal tubules with RCC spheroids under dynamic MPS conditions approximates their physiology to that of the cancer cells. This model represents a novel approach to investigating RCC pathophysiology by replicating aspects of the tumor microenvironment and its interactions with non-tumor cells.

Despite its complexity, our model is still lacking important components of RCC physiology, namely vasculature and a population of immune cells^54^. Incorporating these features in future iterations can benefit the study of anti-angiogenic and immune therapies in vitro. Another limitation of the system is the open nature of the collagen disc incorporating the renal tubule. Although the enhanced polarity and defined basolateral and apical membranes of the tubule these are not compartmentalized. This design precludes Drug metabolism and Pharmacokinetic (DMPK) studies that require de facto separation of the perfusate between the lumen and exterior of the renal tubule, a key requirement for testing drug uptake and excretion. Our approach was also limited to accessing the co-culture effects after 5 days, and prospective studies could expand on this time frame and incorporate supernatant collection at different time points to elucidate the kinetics of immune factor secretion and nutrient consumption.

The use of removable collagen and agar hydrogels compatible with metabolic analysis, fluorescent image acquisition and high content gene expression is a promising approach to evaluating the activity of reconstructed organ structures in MPS. Overall, our microfluidic model illustrates the value of RCC-MPS tools in uro-oncology research. This model can be used to study the impact of RCC metabolism on the efficacy of novel immune therapies, and explore RCC and RPTEC secretions for biomarker discovery applications.

## Materials and methods

### 3D printing

Bespoke culture chambers intended to generate a matrix comprising a reconstructed renal proximal tubule were designed using the open-access computer-assisted design (CAD) software TinkerCAD (Autodesk, San Francisco, CA, USA). The culture chambers were built in-house using small-scale additive manufacturing, commonly known as 3D printing, using a Prusa MK3S+ printer (Prusa Research, Prague, CZ). The bioplastic Polylactic acid (PLA) was chosen as the manufacturing material given its biocompatible and biodegradable properties. Each culture chamber consists of two compartments connected by a microchannel with a diameter of 0.5mm. Each 3D-printed construct comprises two culture chambers laid side by side. The cell compartment has a reverse–cone shape designed to direct the cells into the microchannel. The matrix compartment is rounded (diameter: 8mm, height: 3mm) well and was designed to generate a matrix-shaped in a disc format, compatible with the size of a 96-microplate. A microfilament with a diameter of 0.4mm is inserted into the microchannel, through the culture compartment, and generates a hollow tube after the matrix polymerizes. Design details of the culture chambers are found in supplementary information in Fig. 1 and Table 1, the original stl.file for 3D-printed purposes is also provided.

### Cell culture and MPS preparation

The RPTEC-TERT1, human renal proximal tubule immortalized cell line was obtained from the American Type Culture Collection (ATCC, Manassas, VA, USA) and used as a model of healthy renal tubular epithelium. CAKI-1 cells were obtained from the German Collection of Microorganisms and Cell Cultures (DSMZ, Braunschweig, DE) and used as a model of RCC. Primary human RPTEC were obtained from Biopredic Advancells (Saint-Grégoire, FR) and used also as a model of healthy renal tubular epithelium. All cell lines were cultured using DMEM-12 media supplemented with 10% fetal bovine serum (FBS), 1% Penicillin/Streptomycin, 4 pg/ml Triiodo-L-thyronine, 10 ug/10 ug/10 ng/mL ITS, 32 ng/mL Hydrocortisone, 10 ng/mL human recombinant EGF. Before all assays the cells were maintained in culture at 37 °C, 5% CO_2_ until reaching approximately 90% confluency upon visual inspection.

To generate renal proximal tubules, the culture compartment of the 3D-printed construct was filled with a matrix consisting of low-density rat-tail collagen type I (3–4 mg/mL; Corning Life Sciences, Corning, NY, USA), 1.5 mM of Genipin (30 mM), and 50 mM of NaOH. The matrix-filled chambers were left overnight at 37 °C, 5% CO_2_ to ensure proper polymerization. Subsequently, RPTEC-TERT1 or primary PRPTEC cells were collected and concentrated to a density of 1 million cells/mL and added into the cell compartment of the 3D-printed construct. The microfilament inserted into the connecting microchannel was removed and the cells were allowed to flow into the hollow tube embedded into the collagen matrix. 24 h after adding the cells, the matrixes were removed and kept in static culture in 24-well plates for 5–7 days, and tubule formation was monitored by visual observation. RCC spheroids were generated using hydrogels consisting of 100,000 CAKI-1 cells in 75 uL culture media, 25 µL low-density rat-tail collagen, and 100 µL 2% agarose. The hydrogels were mixed in a 1.5 mL microcentrifuge tube and polymerized at room temperature for 1.5 h. Afterward, the hydrogels were removed from the microtubules and kept in static culture for 4 days. Spheroid formation was monitored by visual observation^41^.

Matured renal tubules and RCC spheroids were cultured under micro-fluidic conditions using the HUMIMIC chip2 MPS platform (TissUse, Berlin, Germany)^36^. The experimental conditions and co-culture combinations are described in Figure X. Renal tubules or RCC spheroid were inserted into each of the culture chambers of the MPS perfusion circuit, along with 250µL of culture media. The chips were perfused at a frequency of 0.25 Hz for 5 days and subsequently, media and cell samples were collected for analysis.

### Morphological characterization

Renal tubule matrices and RCC hydrogels were collected and fixed with 2% paraformaldehyde, followed by permeabilization with 0.1 Triton-X solution in HBSS. To highlight the 3D morphology of the constructs, samples were stained with 1:1000 Hoescht3342 (nucleus) and Phalloidin-488 (f-actin; cytoskeleton) overnight. Imaging was performed using a Keyence BZ-9000 fluorescent microscope or a Leica SP-5 Laser scanning microscope (Wetzlar, Germany). Image processing was completed using the open-access software ImageJ^55^. Details of the immunofluorescent staining’s performed for membrane markers can be found in supplementary table 6.

### Metabolic activity analysis

The Seahorse XF platform (Agilent Technologies, CA, USA), was used to access the cellular bioenergetics of the renal proximal tubules and RCC spheroids after dynamic culture. Assays were performed according to the manufacturer specifications using the Cell Mito Stress Test Kit, to evaluate the oxygen consumption and extracellular acidification rate and infer OxPhos and glycolytic activity. The results obtained were analyzed using the Seahorse companion software Wave.

### Extracellular secretions analysis

Perfusion supernatant samples were collected and stored at – 80 °C prior to analysis. The extracellular levels of IL6, IL8, NGAL, and TNFα were determined using the DuoSet® ELISA kits (R&D Systems, Minneapolis, MN, USA), according to the manufacturer specifications. Samples were diluted at a ratio of 1:200 to optimize detection, and absorbance was acquired using a FLUOstar OPTIMA microplate reader (BMG LabTech, Ortenberg, Germany). The extracellular lactate levels were determined using the bioluminescent Lactate-Glo™ assay (Promega, Madison, WI, USA), according to the manufacturer's specifications.

### RNA extraction and gene expression

The extraction of the RNA from the matrix based on collagen type I (renal tubules) was performed using the RNeasy Mini Kit (Qiagen, Hilden, Germany), according to manufacturer specifications. Each sample consisted of a pool from 3 renal tubule cultures simultaneously. Extraction from the agarose-based hydrogels (RCC spheroids) was performed by initially dissolving the gels in 200 µl Trizol-LS (ThermoFisher, Waltham, MA, USA) and 100 µl chloroform (Sigma-Aldrich, Darmstadt, Deutschland) for 20 min at room temperature and homogenized by resuspending the mixture^56^. For phase separation, an additional 180 µL of chloroform was added and samples centrifuged for 15 min at 4 °C, 15 000 rpm. The aqueous phase was mixed at a 1:2 ratio with isopropanol and centrifuged for 5 min at 15,000 rpm before discarding 1/2 volume and adding 2 × volume of NTC buffer (Macherey–Nagel, Düren, Germany). Samples were incubated for 10 min at 50 °C and subsequently processed using the NucleoSpin Gel and PCR Clean‑up kit (Macherey–Nagel), according to manufacturer specifications. Reverse transcription of purified RNA was performed using the PrimeScript RT Master Mix (Takara, Kusatsu, Japan) according to the specifications. The gene expression of selected markers was analyzed using TaqMan probes (Thermo Fisher) using a C1000 Thermal cycler (BioRad, Hercules, CA, USA). Gene expression data were analyzed using GraphPad Prism 5.00 (GraphPad Software, La Jolla, CA, USA) to determine statistical significance between the different experimental conditions. Experimental groups were compared using a two-tailed unpaired t-test with a confidence interval of 95%. Fold-expression variation analysis was performed based on the ΔΔCt values^57^. The list of primer probes and amplification conditions is described in supplementary information table 2.

### RNA-Seq and bioinformatics analysis

The high-throughput sequencing was performed by NovoGene (Cambridge, UK) including three replicates per treatment (2D and 3D cultures). The sequencing depth was between 12,032,453 and 14,734,138 read-pairs (paired-end sequencing) per sample. The mapping of the reads on the human genome (GRCh38.p14, Ensembl) was performed using NextGenMap^58^. The parameter settings included—very-sensitive and—no-unal beside the standard parameter. In general, ~ 70% of all reads could be mapped on the genome and have been used for gene counting using featureCounts (version 2.0.4)^59^ via the R package. Parameter settings have been adjusted to GTF featureType “gene” and attrType “gene_id” for unstranded reads. The gtf-file was downloaded from the Ensembl (GRCh38.110) and used for the counting. The differentially expressed genes (DEGs) were detected using DESeq2^60^ with standard parameters between 2 and 3D treatment. The DEGs below an adjusted p-value of 0.05 have been split based on their log2foldchange (FC) in 2D upregulated (positive FC) and 2D downregulated (negative FC). Using the R packages complex Heatmap, enhanced Volcano, clusterProfiler, and org.Hs.eg.db led to the identification of overrepresented pathways for GeneOntology (GO) “Biological Process” (BP) and Kyoto Encyclopedia of Genes and Genomes (KEGG) (usage of both databases on 2023-01-02) and the creation of Volcano plots and heatmaps.

### Supplementary Information

Supplementary Information.

## Data Availability

RNA-seq data and DEG analysis can be found in the National Center for Biotechnology Information (NCBI) Gene Expression Omnibus (GEO; https://www.ncbi.nlm.nih.gov/geo/) repository, with the ascension number: GSE244498; token: qhcfocsqhhmjfgp. The .stl file containing the 3D printing specifications of the chamber used to reconstruct the renal tubules can be obtained upon request to the corresponding author.
