# Author Correction: Integrating tumor and healthy epithelium in a micro-physiology multi-compartment approach to study renal cell carcinoma pathophysiology

**DOI:** 10.1038/s41598-024-64959-9

**Published:** 2024-06-25

**Authors:** Maryna Somova, Stefan Simm, Adventina Padmyastuti, Jens Ehrhardt, Janosch Schoon, Ingmar Wolf, Martin Burchardt, Cindy Roennau, Pedro Caetano Pinto

**Affiliations:** 1 Department of Urology, University Medicine Greifswald, DZ7 J05.15, Fleischmannstraße 8, 17475 Greifswald, Germany; 2 Institute of Bioinformatics, University Medicine Greifswald, Fleischmannstraße 8, 17475 Greifswald, Germany; 3 Department of Obstetrics and Gynecology, University Medicine Greifswald, Fleischmannstraße 8, 17475 Greifswald, Germany; 4 Center for Orthopaedics, Trauma Surgery and Rehabilitation Medicine, University Medicine Greifswald, Fleichmannstraße 8, 17475 Greifswald, Germany; 5 Institute for Bioanalysis, Coburg University of Applied Sciences and Arts, Friedrich-Streib-Str. 2, 96450 Coburg, Germany

Correction to: *Scientific Reports* 10.1038/s41598-024-60164-w, published online 23 April 2024

The original version of this Article contained an error in the spelling of the author Jens Ehrhardt which was incorrectly given as Jens Ehrdardt.

The original Article has been corrected.

