# Supplementary material for: Integrating tumor and healthy epithelium in a micro-physiology multi-compartment approach to study renal cell carcinoma pathophysiology
Source: Sci Rep. 2024 Apr 23;14:9357. doi: 10.1038/s41598-024-60164-w (PMC11039668; doi:10.1038/s41598-024-60164-w)
Supplement: Supplementary file 1 — Supplementary Information. [file 41598_2024_60164_MOESM1_ESM.docx]

**
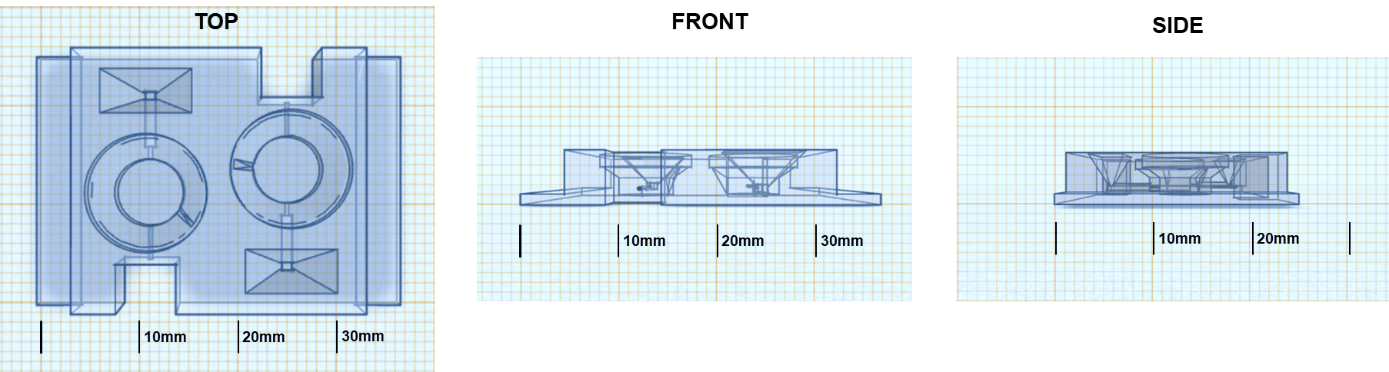
Supplementary Information**

**Supplementary figure 1:** Schematic representation of the cultured chamber designed to generate reconstructed renal tubules and manufactured using 3D printing.

**Supplementary information table 1:** 3D-prining specifications

| Printer | Original Prusa i3 MK3S+ |
| --- | --- |
| Material | Polylactic acid (PLA) |
| Melting temperature | 215°C |
| Base temperature | 60°C |
| Printing resolution | 0.05mm |
| Infilling | 100% |
| Stl.file name | 3D printed chamber Somova et al |

**Supplementary table 2:** Taqman probes used for gene expression analysis

| Gene | Reference | Function |
| --- | --- | --- |
| IL-6 | Hs00174131_m1 | Immune factor |
| IL-8 | Hs00174103_m1 | Immune factor |
| NGAL | Hs01008571_m1 | Immune factor |
| TNFα | Hs00174128_m1 | Immune factor |
| VEGFA | Hs00900055_m1 | Angiogenesis |
| HIF2A | Hs01026149_m1 | Hypoxia |
| HIF1A | Hs00153153_m1 | Hypoxia |
| EGFR | Hs01076090_m1 | Cell growth |
| Nrf-2 | Hs00975961_g1 | Transcription factor |
| TGFβ1 | Hs00998133_m1 | Cell growth |
| OCT1 | Hs00427552_m1 | Drug uptake |
| BCRP | Hs01053790_m1 | Drug efflux |
| P-gp | Hs00184500_m1 | Drug efflux |
| GLUT1 | Hs00892681_m1 | Glucose uptake |
| GAPDH | Hs02786624_g1 | House-keeping |

**Supplementary table 3: ELISA and lactate analysis kits**

| Marker | Supplier | Reference |
| --- | --- | --- |
| IL8 | R&D systems | DY208 |
| NGAL | R&D systems | DY1757 |
| TNFα | R&D systems | DY210 |
| Lactate | Promega | J5021 |

**Supplementary table 4: Agilent Seahorse consumables for metabolic analysis**

| Item | Reference |
| --- | --- |
| Mito Stress test kit | 103010-100 |
| Seahorse XFp FluxPak | 103022-100 |
| XF DMEM medium | 10357-100 |


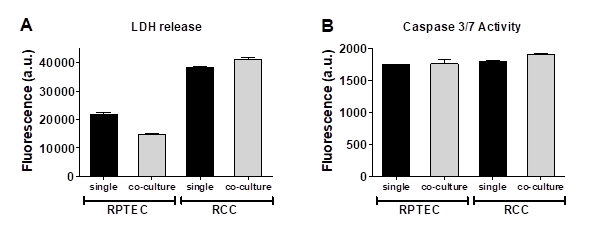


**Supplementary figure 2:** Viability of the renal tubules and RCC spheroids in single and dynamic co-culture. Lactate dehydrogenase (LDH) release after 5 days in culture showed a decrease between single and RCC co-cultured renal tubules **(A)**. The fluorometric assay detects the activity of LDH and extracellular lactate concentrations can act as a limiting step in the fluorescent substrate production. This result is consistent with the absence of lactate in the RPTEC compartment if dynamic culture – Figure 6-D. No LDH differences were overserved between RCC single and co-culture, and overall these results indicate that both the renal tubules and RCC maintain their integrity under the culture conditions tested. The absence of Caspase 3-7 **(B)** activity indicates that the renal tubules and RCC spheroids are not apoptotic under the conditions tested. Moreover, the fluorescent signal retrieved across all conditions is consistent with background levels (about 1500 a.u.) an indication that the dynamic RCC co-culture does not induced cellular stress leading to apoptosis in our experimental set-up.

(LDH assay: LDH-Glo [Promega: J2380] Caspase assay: Caspase 3/7-Glo [Promega: G8090])

**Supplementary table 5:** Statistical analysis of the metabolic activity presented in Figure 7. Statistical significances were determined using a two-tailed unpaired t-test (p < 0.05).

| Basal metabolic rate (%) | | | |
| --- | --- | --- | --- |
| Renal tubule | | | |
| Conditions | Glyco (p-value) | OxPhos (p-value) | Significance |
| 2D *vs* 3D | 0.006 | 0.006 | Yes |
| MPS *vs* Co-culture | 0.025 | 0.025 | Yes |
| RCC | | | |
| Conditions | Glyco (p-value) | OxPhos (p-value) | Significance |
| 2D *vs* 3D | 0.016 | 0.016 | Yes |
| MPS *vs* Co-culture | 0.437 | 0.437 | No |
| ATP production rate (pmol/min) | | | |
| Renal tubule | | | |
| Conditions | Glyco (p-value) | OxPhos (p-value) | Significance |
| 3D *vs* MPS | 0.37 | 0.30 | No |
| MPS *vs* Co-culture | 0.39 | 0.36 | No |
| RCC | | | |
| Conditions | Glyco (p-value) | OxPhos (p-value) | Significance |
| 3D *vs* MPS | 0.07 | 0.19 | No |
| MPS *vs* Co-culture | 0.16 | 0.26 | No |

**Supplementary table 6:** Conditions used for immunofluorescent characterization.

| Primary Ab | Secondary Ab | Dilution | Incubation time | Reference |
| --- | --- | --- | --- | --- |
| EGFR | Alexa-555 | 1:200 /1:500 | 3h /3h | Abcam30 / Invitrogen A21429 |
| ZO1 | - | 1:200 | 3h | Invitrogen 339188 |
|  | | | | |
| Marker | **Dilution/concentration** | **Description** | **Reference** | |
| PNA-lectin-568 | 1:100 | Basement membrane | Invitrogen L32438 | |
| Transferrin-568 | 20ng/mL | Endocytosis ligand | T23365 | |
| Phalloidin-488 | 1:2000 | Cytoskeleton  (f-actin) | Invitrogen A12379 | |
| Hoechst33342 | 20mM / 1:1000 | Nuceli | Merck B2261 | |

**Supplementary figure 3:** Correlation analysis between the 3 biological samples of RPTEC cultured in MPS (3D) and conventional 2D format. The hierarchical clustering shows a consistent expression pattern among the biological samples and a substantial divergence between the 3D and 2D conditions.

**Supplementary figure 4:** Principal component analysis (PCA) of the 3 biological samples of RPTEC cultured in MPS (3D) and conventional 2D format. The PCA plot reiterates the variance between individual biological replicates an the divergence between the 2D and 3D conditions.
